# Mortality Rate and Years of Life Lost due to Hypertension in the South of Iran between 2004 and 2019: A Population-Based Study

**DOI:** 10.1155/2022/7759699

**Published:** 2022-11-29

**Authors:** Alireza Mirahmadizadeh, Mohebat Vali, Jafar Hassanzadeh, Seyed Parsa Dehghani, Ahmadreza Razeghi, Habibollah Azarbakhsh

**Affiliations:** ^1^Non-Communicable Diseases Research Center, Shiraz University of Medical Sciences, Shiraz, Iran; ^2^Student Research Committee, Shiraz University of Medical Sciences, Shiraz, Iran; ^3^Research Center for Health Sciences, Institute of Health, Department of Epidemiology, Shiraz University of Medical Sciences, Shiraz, Iran; ^4^School of Medicine, Shiraz University of Medical Sciences, Shiraz, Iran

## Abstract

**Introduction:**

Hypertension is known worldwide as a preventable significant risk factor for cardiovascular diseases and their mortality. This study was designed to determine the mortality rate and years of life lost (YLL) due to hypertension in Fars Province.

**Method:**

In this cross-sectional study, we extracted all death reports due to hypertension based on age, gender, and the year of death based on ICD-10 from the EDRS system (Electronic Death Registration System). The YLL analysis due to premature death related to hypertension was executed by the 2015 YLL template from WHO in EXCEL 2016 software. To examine the trend of crude and standardized mortality rates and YLL rates for different years, joinpoint regression was used based on the log-linear model.

**Results:**

In the 16 years that the study was done (2004–2019), 13443 death cases occurred in the Fars Province, 51.0% of which (6859 cases) were in females and 48.5% (6515 cases) of which were in the 80+ age group. Total YLL due to hypertension in these 16 years of study was 61,344 (1.9 per 1000) in males, 64,903 (2.1 per 1000) in females, and 126,247 (2.0 in 1000) in both genders. According to the joinpoint regression analysis, the 16-year trend of YLL rate due to premature mortality was increasing: the average annual percent change (AAPC) was 4.9% (95% CI −2.6 to 12.85, *p* value=0.205) for males and 8.4% (95% CI 5.2 to 11.7, *p* value <0.001) for females.

**Conclusion:**

Considering the increasing trend in crude and standardized mortality rates and YLL due to hypertension, it is important for policymakers and decision makers of Health Policy Centers to promote and inform people about the importance of hypertension control and to familiarize them with proper, preventive interventions such as the importance of a healthy diet, routine physical activity, and routine learning programs for different groups in the society especially for people at a higher risk of hypertension.

## 1. Introduction

Hypertension is known worldwide as a preventable significant risk factor for cardiovascular diseases and their mortality. Hypertension is described as systolic blood pressure ≥140 mmHg and diastolic pressure ≥90 mmHg [1]. Hypertension, pre-hypertension, and other blood pressure disorders are responsible for the death of 8.5 million people who die due to ischemic heart diseases, cerebrovascular accidents, and other vascular and renal diseases around the world [2]. As a primary health disorder, hypertension affects 1 billion people worldwide [3].

Although hypertension's prevalence is higher in older people, the burden of disease is potentially higher in younger population, mostly because of increased years of life with disability and a higher chance of acquiring vasculorenal disorders [4].

About two-thirds of hypertension incidence is related to countries with a poor economy. India and China have the highest prevalence of hypertension [5]. The prevalence of hypertension varies significantly in different areas of the world, from 11–30% in Latin American countries to 44% in some regions of Europe [6]. The standardized age-based value of hypertension has increased by 0.08% between 1990 and 2019 [7].

Iran, a developing country with a population of 80 million in the Middle East, has different cultural, socioeconomic backgrounds and lifestyles, all of which are risk factors for hypertension [8].

A meta-analysis study in Iran showed the prevalence of HTN was 25% (95% CI: 19–31) in women and 24% (95% CI: 20–28) in men with no significant difference (*p*=0.758) [9].

Based on the study of the global burden of disease, the age-standardized DALY rate for hypertensive heart disease (HHD) increased by 51.6% for men (95% uncertainty interval (UI) 305.8 to 436.7 per 100,000) and 4.4% for women (95% UI 429.4 to 100,000) in Iran. The age-standardized prevalence of HHD in Iran was almost twice higher than that globally and 1.5 times more than that of the World Bank [10].

The prevalence of hypertension in Iran has been estimated at 31% for adult men and 27% for adult women [11].

Based on a study conducted in Fars Province, the prevalence of hypertension in men and women was 23.8% and 21.1%, respectively, [12]. Therefore, calculating years of life lost (YLL) due to hypertension in Fars Province and describing the current situation can be valuable evidence as base data for policymakers to have a better judgment for their further decisions and interventions. The effect of these interventions can be evaluated and calculated periodically. Therefore, this study was designed to determine the mortality rate and YLL due to hypertension in Fars Province.

## 2. Methods

In this cross-sectional study, carried out in Fars Province between 2004 and 2019, we extracted all death reports due to Hypertension based on age, gender, and the year of death based on ICD-10 from the EDRS system (Electronic Death Registration System). In this study, all deaths due to hypertension-related diseases such as vasculorenal disorders and other abnormalities related to hypertension have been included. The codes executed in this study are l10- l15-. In EDRS, data were gathered from all accessible sources like hospitals, health centers, registry offices, and medical council organizations, and then, the reduplicated death cases were ruled out.

The total population of Fars Province is estimated based on the data from health centers and census done between 1996 and 2016 or with the calculation of annual population growth rate. The standard population in 2013 for low- and intermediate-income countries has been used for standardization [13].

### 2.1. Statistical Analysis

First of all, the crude and age-standardized (ASR) mortality rate of hypertension was calculated based on gender and the year of death.

Then, to calculate YLL, using the table of standard age and determining life expectancy for different age and gender groups, in addition to the number of deaths due to hypertension, calculations were done in each gender and age groups based on the following formula [14]:

(1) $$\begin{array}{c} YLL=\frac{N\;{Ce}^{\left(ra\right)}}{{\left(\beta +r\right)}^{2}}\left[e^{-\left(\beta +r\right)\left(L+a\right)}\left[-\left(\beta +r\right)\left(L+a\right)-1\right]–e^{–\left(\beta +r\right)a}\left[–\left(\beta +r\right)a-1\right]\right]\text{,} \end{array}$$

where *N* is the number of deaths in a specified age and gender group, *L* is the life expectancy of death cases again in that age and gender group, *r* is the discounting rate, which equals 0.03, *β* is a conventional rate in calculating age value which equals 0.04, *C* is an adjusted, constant value that equals 0.1658, *a* is the age on which death occurred, and *e* is a constant value considered as 2.71.

The GBD applied a 3% time discount rate to years of life lost in the future to estimate the net present value of years of life lost.

*β* determines the importance of age-weights.

*C* is an adjustment constant, chosen so that the introduction of age-weights does not alter the total number of years of life lost [15].

In the beginning, YLL due to early death related to hypertension was calculated for 18 age groups (0–4, 5–9–10–15,… and so on up to 85) and was then charted based on the data of the following age groups: 0–4, 5–14, 15–29, 30–44, 45–59, 60–70, 70–79, and 80+.

The YLL analysis due to premature death related to hypertension was executed by the 2015 YLL template from WHO in EXCEL 2016 software.

To examine the trend of crude and standardized mortality rates and YLL rates for different years, joinpoint regression was used based on the log-linear model. Joinpoint regression analysis describes changing trends over successive segments of time and the increase or decrease within each part. The resulting line segment between joinpoints is defined by the annual percent change (APC) based on the slope of the line segment and the average annual percent change (AAPC). Joinpoint Regression Program 4.9.0.0 carried out the analysis for the trend.

The protocol this study was reviewed and confirmed by the Ethics Committee of Shiraz University of Medical Sciences (SUMS) (code: IR.SUMS.REC.1399.772). All aspects of this study were done considering SUMS' ethical code.

## 3. Results

In the 16 years that the study was done (2004–2019), 13443 death cases occurred in the Fars Province, 51.0% of which (6859 cases) were in females and 48.5% (6515 cases) of which were in the 80+ age group.

As shown in Table 1, the crude mortality rate due to hypertension in males increased from 16.0 per 100000 in 2004 to 31.2 per 100000 in 2019 (*p* value for trend = 0.242) and increased from 16.3 per 100000 in 2004 to 32.3 per 100000 in 2019 (*p* value for trend <0.001) in female. The standardized mortality rate in males increased from 21.5 per 100000 in 2004 to 28.8 per 100000 in 2019 (*p* value for trend = 0.205) and increased from 24.1 per 100000 in 2004 to 27.1 in 100000 in 2019 in female (*p* value for trend = 0.001) (Table 1).

Total YLL due to hypertension in these 16 years of study was 61,344 (1.9 per 1000) in males, 64,903(2.1 per 1000) in females, and 126,247 (2.0 in 1000) in both genders. (Gender ratio of female/males equals 1.1) (Table 1).

The average number of years of life lost due to hypertension was 9.31 and 9.46 in males and females, respectively.

**Figure 1:**
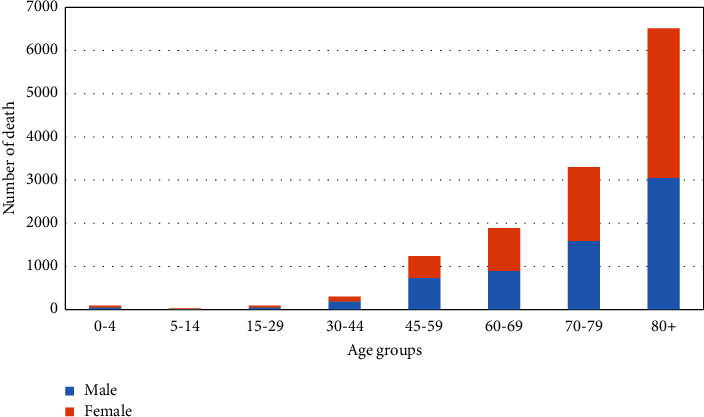
Number of deaths due to hypertension based on gender and age groups.

The most significant number of deaths in both gender group's occurred in the 80+ age group, while the lowest number of deaths in both genders, occurred in the 5–14 age group (Figure 1).

**Figure 2:**
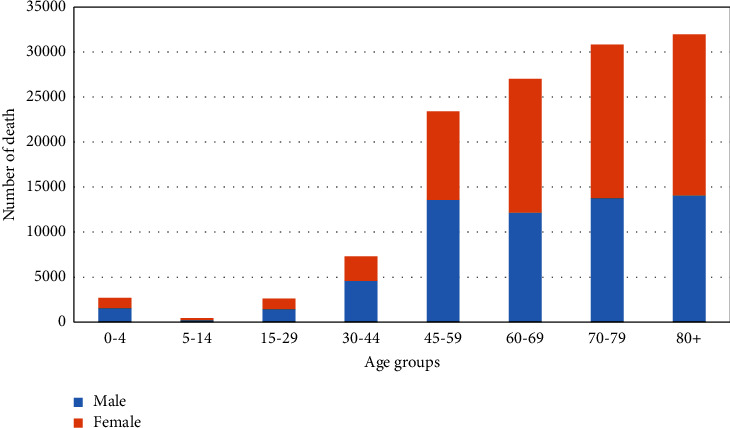
YLL due to hypertension based on gender and age groups.

The most significant YLL in both gender groups occurred in the +80 age group, and the lowest number of YLL in both genders in the 5–14 age group (Figure 2).

**Figure 3:**
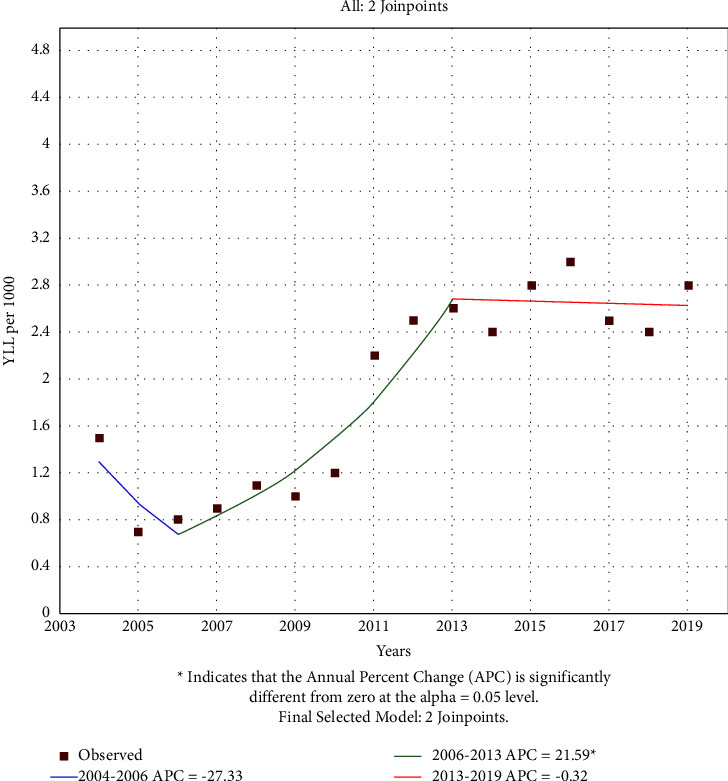
Trend of YLL rate due to hypertension in males 2004–2019.

**Figure 4:**
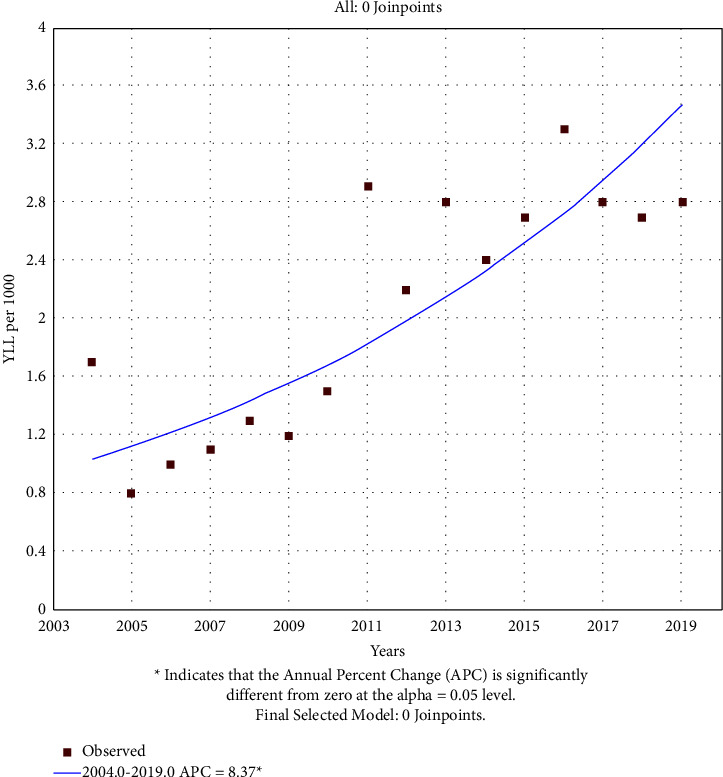
Trend of YLL rate to hypertension in females 2004–2019.

According to the joinpoint regression analysis, the 16 year trend of YLL rate due to premature mortality was increasing: the average annual percent change (AAPC) was 4.9% (95% CI −2.6 to 12.85, *p* value = 0.205) for males, 8.4% (95% CI 5.2 to 11.7, *p* value <0.001) for females (Figures 3 and 4).

The model shows two joinpoints in 2006 and 2013 for males when the APC was −27.3% (95% CI −56.4 to 21.0, *p* value = 0.187) and 21.6% (95% CI 11.5 to 32.5, *p* value = 0.001), respectively.

## 4. Discussion

Hypertension is a major health issue worldwide and accounts for more than one-third of the total deaths and approximately one-fifth of DALY in Europe and Central Asia [16]. Moreover, hypertension is one of the major risk factors for cardiovascular diseases, which has increased constantly worldwide [17].

In this study, 13,443 deaths occurred due to hypertension in the 16 year trend. Our study shows that the burden of hypertension is still considerable, despite modern treatment. The study indicates that standardized and crude rates of death in the 16 years were increasing in both genders. This shows that financial support for managing hypertension has increased on a national scale since 2005; nevertheless, the management of hypertension has not risen significantly compared to developed countries [18, 19]. Therefore, there is room for improvement concerning hypertension management in Iran.

Our study found out that the greatest number of deaths and YLLs belong to the 80+ age group. Hypertension is related to major cardiovascular disorders like ischemic heart failure, cerebrovascular accidents, myocardial infarction, and death in older people. The global burden of hypertension is on the rise due to population aging and an increase in the prevalence of obesity. It is estimated that hypertension will affect one-third of the world's population by 2025 [20]. This evaluation highlights current evidence and emphasizes the recent instructions about hypertension in older adults. Older adults' hypertension management should consider and individualize their degree of weakness, more complex medical disorders, and psychosocial factors. Nonmedical lifestyle medications should be promoted as an adjacent treatment to reduce the need for drug consumption to reduce the risk of acquiring hypertension. Medical therapy with diuretics, RAAS inhibitors, and calcium-channel blockers contributed to older people's cardiovascular disorders. Considering the financial burden of hypertension and its effect on general hygiene worldwide, paying attention to lifestyle modifications in younger generations to prevent hypertension is crucial [20]. Age significantly increases the prevalence of secondary forms of hypertension. This is especially true in patients with atherosclerotic renovascular hypertension, renal failure, and primary hypothyroidism [21].

Changes of YLL in both genders have had an upward trend during the 16 years of study, and this increasing trend continues for females without any changes with a mean annual change that is higher than that of males. While for males, two breakpoints are seen in the trend of YLL in 2006 and 2013. There was a decreasing trend between 2004 and 2006, which changed to an increasing trend between 2006 and 2013; it was somehow constant or slightly decreasing between 2013 and 2019. In addition to the actual incremental trend of YLL and the increasing changes from 2006 to 2013 may be due to increased awareness as well as more screening of blood pressure. In other studies conducted in developed countries, increased blood pressure in women is higher than in men [22]. Gender differences in risk factors and awareness, treatment, and control of hypertension in humans are well established. There are significant differences in the epidemiology and clinical features of hypertension between men and women. In addition, gender differences are associated with certain types of hypertension, including postmenopausal hypertension, white coat hypertension, obscured hypertension, and gestational hypertension disorders. Gender differences play a role in the prevalence and determinants of hypertension and hypertension, while the degree of control is similar between men and women taking antihypertensive drugs. Most importantly, the distinct roles of the angiotensin-converting enzyme 2/Apelin signaling, sex hormone, endothelin-1, and sympathetic neuronal activity contribute to sex differences in blood pressure control [23]. However, like other studies [22, 24–26], low rates of awareness, treatment, and control of blood pressure in Iran indicate the difficulty and inadequate management of this chronic disease at the population level. There are many reasons for inadequate blood pressure control, including poor adherence to treatment, physician inertia, patient's initial cardiovascular risk, and poor adherence to guidelines. Also, physicians' criteria for diagnosing and determining high BP may be highly subjective and done regardless of the requirements specified by regional and international management guidelines [22].

Our study has limitations: the different quality of data gathered during the 16 years. Other factors such as awareness-raising and screening programs like the National Blood Pressure Control Mobilization may have also caused the difference in these 16 years. Differences in the definition of blood pressure can also be another reason for this incongruity. Nevertheless, this is a population-based study and analyzes a significant sample volume; therefore, it helps policymakers better judge their policies and interventions. It is suggested that in future studies, different definitions of blood pressure be considered to examine the mortality rate and years of life lost due to hypertension. It is also suggested that future studies consider the impact of various variables such as diet changes, training, or the impact of health campaigns on the mortality rate and years of life lost due to hypertension.

## 5. Conclusion

Considering the increasing trend in crude and standardized mortality rate and YLL due to hypertension, it is important for policymakers and decision makers of Health Policy Centers to promote and inform people about the importance of hypertension control and to familiarize them with proper, preventive interventions such as the importance of a healthy diet, routine physical activity, routine learning programs for different groups in the society especially for people at a higher risk of hypertension, and routine measurements of blood pressure.

## Figures and Tables

**Table 1:** Crude and standardized mortality rates (per 100,000 population) and YLL due to hypertension based on gender and year in Fars Province 2004–2019.

Year	*No. of deaths*		*Crude mortality rate*		*ASR (95%CI)*		*YLL*			
							*No.*		*(per 1000)*	
	Male	Female	Male	Female	Male	Female	Male	Female	Male	Female
2004	298	289	16.0	16.3	21.5 (19.6–23.3)	24.1 (22.3–26.0)	2850	3079	1.5	1.7
2005	149	145	8.4	8.2	10.6 (9.3–11.8)	11.2 (9.9–12.6)	1384	1496	0.7	0.8
2006	156	174	8.4	9.6	10.5 (9.1–11.8)	12.5 (11.1–14.0)	1479	1752	0.8	1.0
2007	165	190	8.8	10.4	10.7 (9.3–12.0)	12.8 (11.3–14.3)	1604	1950	0.9	1.1
2008	240	250	12.7	13.5	13.8 (12.2–15.4)	15.0 (13.3–16.7)	2144	2484	1.1	1.3
2009	201	217	10.6	11.6	11.2 (9.7–12.6)	13.5 (12.0–15.0)	1961	2271	1.0	1.2
2010	236	254	12.3	13.4	12.4 (10.8–13.9)	14.0 (12.4–15.7)	2285	2789	1.2	1.5
2011	476	576	24.5	29.9	23.3 (21.2–25.6)	29.4 (27.0–31.9)	4338	5523	2.2	2.9
2012	403	343	20.5	17.6	20.3 (18.3–22.3)	18.1 (16.2–20.0)	5000	4270	2.5	2.2
2013	610	627	30.6	31.9	27.9 (25.5–30.4)	29.6 (27.1–32.1)	5317	5591	2.6	2.8
2014	530	516	26.2	26.0	24.2 (22.0–26.4)	23.8 (21.5–26.0)	4942	4851	2.4	2.4
2015	621	594	30.3	29.6	27.3 (24.9–29.7)	27.3 (24.9–29.7)	5676	5487	2.8	2.7
2016	689	743	33.2	36.6	29.8 (27.3–32.3)	32.4 (29.7–35.0)	6135	6597	3.0	3.3
2017	597	681	28.7	33.6	25.8 (23.5–28.1)	30.31 (27.8–32.8)	5232	5747	2.5	2.8
2018	557	600	26.6	29.5	23.9 (21.7–26.1)	26.1 (23.8–28.5)	5000	5386	2.4	2.7
2019	656	660	31.2	32.3	28.8 (26.4–31.2)	27.1 (24.6–29.6)	5997	5630	2.8	2.8
Total	6584	6859	20.9	22.3	21.2 (20.7–21.7)	22.9 (22.4–23.5)	61344	64903	1.9	2.1
*p* value	—	—	0.242	<0.001	0.205	0.001	—	—	0.205	<0.001

## Data Availability

The data for the current study will not be shared publicly.
